# Supplementary material for: 6′-Sialyllactose Ameliorates In Vivo and In Vitro Benign Prostatic Hyperplasia by Regulating the E2F1/pRb–AR Pathway
Source: Nutrients. 2019 Sep 12;11(9):2203. doi: 10.3390/nu11092203 (PMC6770807; doi:10.3390/nu11092203)
Supplement: Supplementary file 1 [file nutrients-11-02203-s001.pdf]

Supplementary data

Supplementary data 1.

A. Prostate weight (mg)

Prostate weight (mg)					
	**CON**	**BPH**	**Fina**	**6SL 0.5**	**6SL 1.0**
1	104.5	975	855.9	755.5	833.2
2	129.7	970	814.9	622.5	770.4
3	145.6	851	755.7	794.9	686.7
4	99.5	867.9	786.5	653.8	670.1
5	152.5	1042	805.4	689.9	797.1
6	101.9	1033.1	770.8	561.4	829.1
7	142.8	918.7	866.5	562.7	891.9
8	121.5	829	638.7	586	790.9

B. PW/BW (mg/g)

Prostate weight/Body weight (PW/BW, mg/g)					
	**CON**	**BPH**	**Fina**	**6SL 0.5**	**6SL 1.0**
1	0.310089	3.037383	2.778896	2.489292	2.838842
2	0.370043	3.104	2.586984	1.844444	2.434123
3	0.441212	2.701587	2.605862	2.523492	2.339693
4	0.292647	2.633991	2.67517	1.966316	2.168608
5	0.42777	3.372168	2.767698	2.269408	2.725128
6	0.299706	3.749909	2.390078	1.846711	2.782215
7	0.391233	2.678426	2.690994	1.988339	3.054452
8	0.357353	2.648562	1.974343	1.691198	2.463863

C. Concentration of DHT (ng/mL)

Concentration of DHT (ng/mL)					
	**CON**	**BPH**	**Fina**	**6SL 0.5**	**6SL 1.0**
1	0.07956	6.22156	5.22186	5.12186	4.77658
2	0.092	6.05984	4.8191	4.9191	4.85448
3	0.07908	6.22156	5.07658	5.07658	5.02136
4	0.06964	6.35984	5.05449	5.05449	4.8193

Supplementary data 2.

A. TETP (μm)

	TETP (μm)				
	**CON**	**BPH**	**Fina**	**6SL 0.5**	**6SL 1.0**
1	12.52	62.06	36.57	38.57	29.52
2	16.02	64.22	35.22	36.57	30.98
3	14.95	68.57	40.98	36.05	29.57
4	11.03	60.56	36.05	38.23	27.51
5	13.77	61.34	34.45	40.05	33.34

B. Relative protein level of AR, PSA and PCNA

		**1**	**2**	**3**	**Average**	**STDEV**
**AR/β-actin**	Con	0.143489	0.130418	0.136943	0.13695	0.006535
	BPH	1.005648	0.994375	0.999977	1	0.005637
	Fina	0.02178	0.021419	0.021601	0.0216	0.000181
	6SL 0.5	0.018962	0.015576	0.017269	0.017269	0.001693
	6SL 1.0	0.023548	0.019263	0.021402	0.021404	0.002143
**PSA/β-actin**	Con	0.027681	0.02537	0.026527	0.026526	0.001156
	BPH	0.997591	1.002411	0.999998	1	0.00241
	Fina	0.025516	0.02531	0.025414	0.025413	0.000103
	6SL 0.5	0.004065	0.005058	0.004562	0.004562	0.000496
	6SL 1.0	0.003217	0.001611	0.002405	0.002411	0.000803
**PCNA/β-actin**	Con	0.082634	0.079834	0.081249	0.081239	0.0014
	BPH	0.998171	1.001823	1.000006	1	0.001826
	Fina	0.35089	0.34736	0.349147	0.349132	0.001765
	6SL 0.5	0.654035	0.67149	0.662682	0.662736	0.008728
	6SL 1.0	0.648875	0.658303	0.653566	0.653582	0.004714

Supplementary data 3.

A. Relative protein level of p-Rb and E2F1

		1	2	3	Average	STDEV
	Con	0.738378	0.724048	0.731167	0.731198	0.007165
	BPH	1.00202	0.997988	0.999992	1	0.002016
**p-Rb/β-actin**	Fina	0.655888	0.657941	0.656918	0.656916	0.001026
	6SL 0.5	0.755866	0.759546	0.757689	0.7577	0.00184
	6SL 1.0	0.817904	0.837647	0.827731	0.827761	0.009871
	Con	0.168307	0.195236	0.181701	0.181748	0.013465
	BPH	0.992808	1.007221	0.999971	1	0.007206
**E2F1/β-actin**	Fina	0.222529	0.225437	0.223996	0.223987	0.001454
	6SL 0.5	0.152795	0.162743	0.157804	0.157781	0.004974
	6SL 1.0	0.13951	0.137794	0.138662	0.138655	0.000858

B. Relative protein level of Cyclin A, Cdk2, Cyclin D1 and Cdk6

		1	2	3	Average	STDEV
	Con	0.014724	0.016188	0.01544	0.015451	0.000732
	BPH	0.974503	1.02605	0.999446	1	0.025778
**Cyclin A/β-actin**	Fina	0.022928	0.013648	0.018272	0.018283	0.00464
	6SL 0.5	0.026949	0.027262	0.027102	0.027104	0.000156
	6SL 1.0	0.040887	0.037225	0.039024	0.039045	0.001831
	Con	0.016609	0.017944	0.017285	0.017279	0.000667
	BPH	1.013841	0.986295	0.999864	1	0.013773
**Cdk2/β-actin**	Fina	0.239212	0.250837	0.245156	0.245069	0.005813
	6SL 0.5	0.706553	0.681677	0.693938	0.694056	0.012438
	6SL 1.0	0.260556	0.232279	0.246132	0.246322	0.014139
	Con	0.142248	0.137735	0.140022	0.140002	0.002256
	BPH	0.981202	1.018977	0.999821	1	0.018888
**Cyclin D1/β-actin**	Fina	0.83125	0.812411	0.82183	0.82183	0.009419
	6SL 0.5	0.541033	0.575316	0.557923	0.558091	0.017142
	6SL 1.0	0.372308	0.375464	0.373912	0.373895	0.001578
	Con	0.065731	0.066908	0.066321	0.06632	0.000589
	BPH	1.005527	0.994487	0.999986	1	0.00552
**Cdk6/β-actin**	Fina	0.198023	0.203469	0.200715	0.200736	0.002723
	6SL 0.5	0.504562	0.494734	0.499601	0.499632	0.004914
	6SL 1.0	0.460078	0.456271	0.458181	0.458177	0.001903

Supplementary data 4.

A. MTT assay

6SL (μM)	1	2	3	4	5	6	Average	STDEV
**DMSO**	95.89928	99.20863	101.0791	101.0791	99.92806	102.8058	100	2.351539
**3.125**	89.4964	90.28777	106.1151	91.51079	92.66187	93.30935	93.89688	6.152054
**6.25**	88.20144	93.16547	92.30216	92.15827	90.5036	91.29496	91.27098	1.75791
**12.5**	94.17266	92.94964	99.56835	99.4964	98.1295	98.27338	97.09832	2.830793
**25**	95.03597	101.0791	104.6043	109.8561	99.35252	98.63309	101.4269	5.181138
**50**	89.20863	88.99281	98.48921	89.92806	98.1295	95.17986	93.32134	4.482059
**100**	79.71223	82.73381	88.77698	89.13669	88.48921	88.92086	86.29496	4.048705
**200**	75.03597	79.78417	76.04317	84.82014	77.05036	79.64029	78.72902	3.541634

B. Relative protein level of AR and PSA

		1	2	3	Average	STDEV
**AR/β-actin**	-	1.006062	0.993963	0.999976	1	0.00605
	6SL 12.5	0.525118	0.51865	0.521892	0.521887	0.003234
	6SL 25	0.401662	0.416059	0.40877	0.408831	0.007199
	6SL 50	0.15763	0.158329	0.157982	0.15798	0.00035
**PSA/β-actin**	-	0.992419	1.007577	1.000004	1	0.007579
	6SL 12.5	0.121689	0.107801	0.114832	0.114774	0.006944
	6SL 25	0.225937	0.209017	0.217298	0.217417	0.008461
	6SL 50	0.061443	0.068214	0.06483	0.064829	0.003386

Supplementary data 5.

A. Relative protein level of p-Rb and E2F1

		1	2	3	Average	STDEV
**p-Rb/β-actin**	-	1.022446	0.977635	0.99992	1	0.022405
	6SL 12.5	0.479141	0.435467	0.4573	0.457303	0.021837
	6SL 25	0.507523	0.47675	0.492205	0.49216	0.015387
	6SL 50	0.380629	0.353555	0.367079	0.367088	0.013537
**E2F1/β-actin**	-	0.9902	1.009806	0.999995	1	0.009803
	6SL 12.5	0.319763	0.306186	0.312945	0.312965	0.006789
	6SL 25	0.353403	0.355337	0.354375	0.354372	0.000967
	6SL 50	0.164321	0.166728	0.165513	0.165521	0.001203

B. Relative protein level of Cyclin A, Cdk2 and Cyclin D1

		1	2	3	Average	STDEV
**Cyclin A/β-actin**	-	1.312585	0.742346	0.945069	1	0.289061
	6SL 12.5	0.895574	0.741525	0.810681	0.815927	0.077159
	6SL 25	0.834412	0.727465	0.777179	0.779686	0.053518
	6SL 50	0.548709	0.750237	0.633269	0.644072	0.101197
**Cdk2/β-actin**	-	0.990932	1.009078	0.99999	1	0.009073
	6SL 12.5	0.794188	0.800509	0.797345	0.797347	0.00316
	6SL 25	0.908676	0.883768	0.896052	0.896165	0.012454
	6SL 50	0.45015	0.465889	0.45792	0.457986	0.00787
**Cyclin D1/β-actin**	-	1.181475	0.819895	0.998629	1	0.180794
	6SL 12.5	1.561353	1.497086	1.528981	1.52914	0.032133
	6SL 25	1.390017	1.106845	1.250778	1.249213	0.141593
	6SL 50	0.585695	0.239658	0.410908	0.412087	0.173022
